# The overestimated prevalence of hypertension in a population survey: a cross-sectional study from Hebei province, China

**DOI:** 10.1186/s12872-022-02994-y

**Published:** 2022-12-12

**Authors:** Xue Geng, Yaqing Zhou, Xiaoli Gao, Feng Li, Guoqiang Gu, Long Bai, Wei Cui

**Affiliations:** 1 Department of Cardiology, The Second Hospital of Hebei Medical University and Institute of Cardiocerebrovascular Disease of Hebei Province, No. 215, He Ping West Road, 050000 Shijiazhuang, China; 2 Department of Cardiology, North China Petroleum General Hospital, Cangzhou, China; 3 Department of Quality Control, The Second Hospital of Hebei Medical University, Shijiazhuang, China

**Keywords:** Hypertension prevalence, Population surveys, One-visit survey, Ambulatory blood pressure monitoring, Daytime multiple visit survey

## Abstract

**Objective:**

Currently, the prevalence of hypertension is mainly ascertained using a one-visit population survey, which may lead to overestimation. The purpose of this study was to assess the accuracy of hypertension prevalence determined by a one-visit population survey.

**Methods:**

For this cross-sectional study, we continuously enrolled 1116 volunteers without a hypertension history in Hebei province from January 2018 to December 2019. The study population included 511 (45.80%) males and 605 (54.20%) females with a mean age of 48 years. The hypertension prevalence was assessed using two methods: one-visit screening and daytime ambulatory blood pressure (BP) monitoring. We directly compared the performances of daytime ambulatory BP monitoring and one-visit screening in the same group of subjects. In addition, we explored possible thresholds to improve the detection of hypertension.

**Results:**

During the one-visit survey, the mean BP value was about 8 mmHg higher than that determined by daytime ambulatory BP monitoring. The prevalence of hypertension was 29.84% and 14.07% during the one-visit and daytime multiple visit surveys, respectively. The risk factors for overestimated hypertension were female sex, body mass index < 24.00 kg/m^2^, and diastolic BP of 100 mmHg. The positive predictive value of the one-visit population survey for diagnosing hypertension was 36.34%. Furthermore, receiver operating characteristic analysis showed that in males, the best diagnostic threshold for hypertension diagnosis was 148/96 mmHg.

**Conclusion:**

The hypertension prevalence was likely overestimated by 2-fold in the one-visit survey group compared to the daytime ambulatory BP monitoring group. Thus, the threshold for one-visit BP screening should be raised to 148/96 mmHg to improve the accuracy of hypertension diagnosis.

**Supplementary Information:**

The online version contains supplementary material available at 10.1186/s12872-022-02994-y.

## Introduction

Hypertension is the leading risk factor for cardiovascular and cerebrovascular diseases, and thus is a serious threat to human health. The prevalence of hypertension has been increasing worldwide [1]. During May Measurement Month 2019, the global prevalence of hypertension was 30.4% [2], whereas the global overall prevalence of hypertension in adults is approximately 30–45% [3]. Kearney et al. [4] estimated that the global hypertensive population will increase by 15–20% by 2025. The prevalence of hypertension in the Chinese population is also high (30.54%) and significantly increased after applying the 2017 American College of Cardiology/American Heart Association Updated Hypertension Guidelines [5]. However, there has been little research into whether current epidemiological screening methods accurately reflect the prevalence of hypertension.

May Measurement Month is a global blood pressure (BP) screening campaign to assess the worldwide prevalence of hypertension [2, 6, 7]. In this screening campaign, BP is measured three times continuously during a single interview; however, the BP is characterized by variability that reflects the subject’s hemodynamic state [8]. BP varies with many factors such as physical activity, time of the day, season of the year, stress, and environmental changes. For this reason, BP measured during one-visit screening is likely to be inaccurate and overestimate the actual prevalence of hypertension, because it does not take into account variability and often mistakes an occasional increase in BP for hypertension [9–11]. An additional reason for overestimation could be white coat hypertension, which is characterized by BP that is only high in the doctor’s office. The overestimation of hypertension prevalence in epidemiological surveys should raise attention; however, very few studies have comprehensively investigated the prevalence of overestimation and ways to improve the situation.

It is generally accepted that 24-h ambulatory BP monitoring (24-h ABPM) is the most accurate way to measure BP, as it has better sensitivity and specificity than clinical or home BP measurements for screening and diagnosing hypertension [12, 13]. Many guidelines recommend using 24-h ABPM as the main way to screen or diagnose hypertension [14–22]. Daytime ABPM (D-ABPM) is measured when individuals are awake and can provide a more representative BP level. Compared with 24-h ABPM, D-ABPM only needs to be monitored during the daytime and may be better accepted by patients. D-ABPM can distinguish between low- and high-risk patients with hypertension and is a better predictor of cardiovascular and cerebrovascular disease prognostication than screening BP [23–26].

A few studies have employed D-ABPM to determine the prevalence of hypertension. In this study, the prevalence of hypertension was simultaneously measured by D-ABPM and a one-visit survey. This study also explored the predictive value of BP level in a one-visit population survey compared to true positive hypertension in order to provide a basis for the accurate estimation of hypertension prevalence.

## Materials and methods

### Study participants

This study continuously recruited 1352 participants without a history of hypertension from January 2018 to December 2019 in Hebei province. All participants underwent two BP measurements: D-ABPM and one-visit survey. This study was approved by the ethics committee of the Second Hospital of Hebei Medical University (2021-P034), and all participants provided written informed consent and volunteered to participate in the study.

Subjects who had no history of hypertension, provided informed consent, volunteered to undergo D-ABPM, and were ≥ 18 years of age were included in the study. The exclusion criteria were: primary or secondary hypertension, acute myocardial infarction, stroke or other acute severe diseases, recent usage of hormonal drugs interfering with BP, unexplained abnormal BP, or D-ABPM time less than 24 h.

## Study method

### Study process

This study was performed by a cardiovascular physician for clinical data collection (e.g., basic information, past medical history, personal history, family history), physical examination, laboratory examination, electrocardiogram, BP measurement during the one-visit survey, D-ABPM, and other auxiliary examinations.

### BP measurement in the one-visit survey group

An Omron medical electronic sphygmomanometer (HEM-8102; Omron, Kyoto, Japan) was selected as the BP measuring instrument. The measurement of BP was performed under supervision of competent medical personnel. Subjects were not allowed to smoke or drink caffeinated beverages 30 min before the examination. After 5 min of quiet rest, subjects were asked to expose their right upper arm. The middle of the airbag was aligned with the brachial artery. BP was continuously measured three times, and the interval between each measurement was 1–2 min. The average BP of the second and third readings was used as the final result.

### BP measurement in the D-ABPM group

D-ABPM was monitored using the CONTEC ambulatory BP monitor (ABPM50; Qinhuangdao Kangtai Medical System, Qinhuangdao, China). The BP monitoring protocol was performed in a series of steps. First, a suitable upper arm was selected for monitoring. The BP of the left and right arms was measured in the clinic. If the difference was > 10 mmHg, the arm with higher BP was selected to monitor the D-ABPM. If the difference was < 10 mmHg, the non-dominant arm was used. Second, the appropriate cuff was chosen, followed by installation of the instrument. Third, BP was manually measured twice to ensure that the instrument was functioning normally. The subjects were instructed to carry out daily activities but avoid intense exercise, driving, showering, and swimming during monitoring. They were told to keep still and let their upper arm droop during BP measurements. Subjects were reminded that BP measurement may cause discomfort or affect sleep. BP was measured every 30 min during waking hours (07:00–22:00).

### Description and diagnosis of different indicators

BP was measured consecutively three times during the one-visit survey. The average BP of the last two readings was used as the BP measurement results of the one-visit survey. Systolic BP (SBP) ≥ 140 mmHg and/or diastolic BP (DBP) ≥ 90 mmHg were used as a cutoff to diagnose hypertension [20]. Daytime ambulatory SBP (D-ASBP) ≥ 135mmHg and/or daytime ambulatory DBP (D-ADBP) ≥ 85mmHg were used as a cutoff to diagnose daytime ambulatory hypertension [12, 20, 27]. Hypertension prevalence rate was defined as the proportion of subjects with hypertension to the total number of individuals. Hypertension prevalence rate in the one-visit survey was defined as the percentage of subjects with SBP/DBP ≥ 140/90 mmHg. Daytime ambulatory hypertension prevalence rate was defined as the percentage of subjects with D-A SBP/D-ADBP ≥ 135/85 mmHg.

Regarding the positive predictive value (PPV) of BP in the one-visit population survey: BP ≥ 140/90 mmHg in the one-visit survey was considered positive for screening and D-ABP ≥ 135/85 mmHg was used as the diagnostic criteria for hypertension. The PPV was estimated, namely the possibility of hypertension in the population with elevated BP was measured in the one-visit survey.

Study participants were divided into four groups according to their body mass index (BMI): low (< 18.50 kg/m^2^), normal (≥ 18.50 to < 24.00 kg/m^2^), overweight (≥ 24.00 to < 28.00 kg/m^2^), and obesity (≥ 28.00 kg/m^2^) [28]. In addition, subjects were divided into three groups according to their cholesterol (CHOL) levels: low (< 3.11 mmol/L), normal (≥ 3.11 to < 5.20 mmol/L), and high (≥ 5.20 mmol/L).

### Statistical analyses

SPSS version 25.0 (SPSS, Chicago, IL, USA) was used for statistical analyses. Data were tested for normal distribution using the Shapiro-Wilk test and are shown as the mean ± standard deviation in accordance with normal distribution. The comparison of BP levels was performed using the independent samples *t*-test for two groups or analysis of variance for more than two groups. The hypertension prevalence and PPV are expressed as percentage (%). The Kappa consistency test and McNemar test were used to compare the prevalence between daytime ambulatory hypertension and the one-visit survey. Baseline analyses of the overestimated population were performed with the chi-square test or Fisher’s exact probability method. Multivariable logistic regression was applied to assess risk factors of the overestimated population. Receiver operating characteristic (ROC) was used to calculate the best diagnostic threshold for predicting true positive hypertension. The area under the ROC curve (AUC) was analyzed using MEDCALC software. *P* < 0.05 was considered statistically significant.

## Results

### Analyses of BP using the one-visit survey or D-ABPM

This study continuously recruited 1352 participants without a hypertension history from January 2018 to December 2019. However, 236 participants were excluded from the study for several reasons, including BP measurement time that was too long or too short, effective measurement times that were not according to a standard, effective rate less than 70%, or incomplete data. Finally, 1116 cases were included in the study including 511 males (45.80%) and 605 females (54.20%), with a mean age of 48 (18–84 years). (See the Additional file 1: Figure S1). Both SBP and DBP measured in the one-visit survey group were significantly higher than that in the D-ABPM group, with the exception of DBP in patients with chronic renal disease (*P* < 0.05). The mean BP in the one-visit survey group was approximately 8 mmHg higher than that of the D-ABPM group. In addition, SBP and DBP in both the one-visit survey and D-ABPM groups were significantly different across subjects with differences in sex, age, BMI, occupation, CHOL status, smoking and drinking history (*P* < 0.05) (Table 1).

**Table 1:** The sociodemographic characteristics and blood pressure level of different screening and measurement patterns (N = 1116)

Parameters	N (%)	D-ABPM (Mean ± SD)		BP of one-visit survey (Mean ± SD)	
		SBP	DBP	SBP	DBP
Total	1116 (100.00%)	118.44 ± 11.60	74.41 ± 8.85	127.34 ± 16.94^†^	81.24 ± 11.84^†^
*Sex*					
Male	511 (45.80%)	121.91 ± 10.99*	77.99 ± 8.80*	130.54 ± 16.30 *^†^	84.73 ± 11.66 *^†^
Female	605 (54.20%)	115.50 ± 11.29	71.38 ± 7.70	124.63 ± 17.02 ^†^	78.29 ± 11.18 ^†^
*Age groups, (years)*					
≤ 44	464 (41.60%)	115.52 ± 11.19*	73.54 ± 9.45*	123.56 ± 15.89 *^†^	80.03 ± 11.99 *^†^
45–59	416 (37.30%)	119.67 ± 11.21	76.34 ± 8.42	127.89 ± 15.99 ^†^	83.38 ± 11.45 ^†^
60–74	219 (19.60%)	121.49 ± 11.66	72.77 ± 7.70	133.07 ± 18.20 ^†^	80.02 ± 11.79 ^†^
≥ 75	17 (1.50%)	128.39 ± 11.00	72.00 ± 8.37	143.00 ± 20.44 ^†^	77.47 ± 10.70 ^†^
*BMI groups, (kg/m*^2^)^#^					
< 18.50	19 (1.70%)	108.11 ± 10.34*	68.41 ± 9.48*	112.00 ± 8.93* ^†^	74.74 ± 10.76 *^†^
≥ 18.50 ~ < 24.00	467 (41.80%)	115.21 ± 10.72	71.65 ± 7.77	124.29 ± 17.78 ^†^	78.18 ± 11.64 ^†^
≥ 24.00 ~ < 28.00	438 (39.20%)	120.28 ± 11.78	76.04 ± 8.93	128.95 ± 15.96 ^†^	82.97 ± 11.32 ^†^
≥ 28.00	190 (17.00%)	123.08 ± 10.57	78.09 ± 8.80	132.44 ± 15.31 ^†^	85.36 ± 11.56 ^†^
*Occupation*					
Physical laborer	403 (36.10%)	119.06 ± 11.37*	74.88 ± 8.99*	129.07 ± 16.74 *^†^	82.28 ± 12.27 *^†^
Staff	449 (40.20%)	117.13 ± 11.31	74.86 ± 9.03	124.88 ± 16.39 ^†^	81.45 ± 11.61 ^†^
Retirees	135 (12.10%)	122.32 ± 12.60	72.71 ± 7.16	133.17 ± 18.90 ^†^	79.47 ± 10.90 ^†^
Freelance	129 (11.60%)	116.96 ± 11.24	73.14 ± 9.15	124.37 ± 15.20 ^†^	79.08 ± 11.86 ^†^
*Smoking*					
No	799 (71.60%)	116.90 ± 11.26*	72.86 ± 8.23*	125.81 ± 16.69 *^†^	79.50 ± 11.17 *^†^
Yes	317 (28.40%)	122.31 ± 11.54	78.32 ± 9.16	131.19 ± 16.98 ^†^	85.61 ± 12.36 ^†^
*Drinking*					
No	720 (64.50%)	116.91 ± 11.55*	72.63 ± 8.41*	126.34 ± 16.85 *^†^	79.68 ± 11.46* ^†^
Yes	396 (35.50%)	121.22 ± 11.17	77.64 ± 8.73	129.16 ± 16.99 ^†^	84.07 ± 12.00 ^†^
*Hyperlipidemia history*					
No	1042 (93.40%)	118.26 ± 11.43	74.44 ± 8.91	127.30 ± 17.05 ^†^	81.33 ± 11.88 ^†^
Yes	74 (6.60%)	120.91 ± 13.59	73.91 ± 8.11	127.91 ± 15.46 ^†^	79.99 ± 11.33 ^†^
*Diabetes history*					
No	1066 (95.50%)	118.22 ± 11.42*	74.39 ± 8.89	127.23 ± 16.94 ^†^	81.28 ± 11.95 ^†^
Yes	50 (4.50%)	123.13 ± 14.17	74.75 ± 8.11	129.68 ± 16.99 ^†^	80.38 ± 9.30 ^†^
*Cardiovascular history*					
No	1096 (98.20%)	118.40 ± 11.54	74.44 ± 8.85	127.30 ± 16.83 ^†^	81.27 ± 11.80 ^†^
Yes	20 (1.80%)	120.33 ± 14.87	72.64 ± 9.05	129.35 ± 22.93 ^†^	79.25 ± 13.98 ^†^
*Cerebrovascular history*					
No	1101 (98.70%)	118.40 ± 11.56	74.42 ± 8.88	127.24 ± 16.83 ^†^	81.22 ± 11.87 ^†^
Yes	15 (1.30%)	121.38 ± 14.26	73.69 ± 7.24	134.53 ± 23.25 ^†^	82.80 ± 9.07 ^†^
*Respiratory history*					
No	1094 (98.00%)	118.41 ± 11.53	74.46 ± 8.80	127.29 ± 16.81 ^†^	81.24 ± 11.84 ^†^
Yes	22 (2.00%)	119.92 ± 14.88	71.85 ± 11.05	129.73 ± 22.96 ^†^	80.86 ± 11.91 ^†^
*Chronic renal damage history*					
No	1113 (99.70%)	118.39 ± 11.52	74.40 ± 8.85	127.25 ± 16.83* ^†^	81.23 ± 11.85 ^†^
Yes	3 (0.30%)	137.33 ± 25.48	77.33 ± 10.60	161.67 ± 27.97 ^†^	83.00 ± 9.85
*CHOL groups, (mmol/L)*^#^					
< 3.11	377 (33.80%)	116.21 ± 10.87*	73.05 ± 8.71*	125.46 ± 17.11 *^†^	80.02 ± 11.81 *^†^
≥ 3.11 ~ < 5.20	495 (44.40%)	118.51 ± 11.97	74.77 ± 8.94	127.18 ± 16.59 ^†^	81.31 ± 11.60 ^†^
≥ 5.20	182 (16.30%)	121.74 ± 11.30	76.11 ± 8.61	131.40 ± 17.07 ^†^	83.91 ± 12.70 ^†^
*eGFRgroups,(ml/min/1.73m*^2^)^#^					
≥ 90	871 (78.00%)	117.98 ± 11.34	74.65 ± 8.81	126.70 ± 16.68 *^†^	81.44 ± 12.02 ^†^
< 90	166 (14.90%)	119.65 ± 12.9	73.31 ± 8.99	130.35 ± 18.23 ^†^	80.66 ± 11.79 ^†^
*Four results of one-visit hypertension*					
True negative	747 (66.90%)	113.52 ± 8.08*	70.88 ± 6.53*	118.86 ± 10.80 *^†^	76.02 ± 7.96* ^†^
False positive	212 (19.00%)	121.55 ± 6.40	76.42 ± 5.75	144.05 ± 12.39 ^†^	89.85 ± 10.45 ^†^
False negative	36 (3.20%)	133.16 ± 7.84	85.82 ± 3.32	129.61 ± 5.67	82.69 ± 5.12 ^†^
True positive	121 (10.8%)	138.96 ± 9.83	89.27 ± 7.46	149.72 ± 14.73 ^†^	97.94 ± 11.22 ^†^

BMI indicates body mass index; CHOL indicates cholesterol; eGFR indicates estimation of glomerular filtration rate; BP indicates blood pressure; D-ABPM indicates daytime ambulatory blood pressure

*Presents difference within different groups, *P* < 0.05

†Presents difference between D-ABPM and BP of one-visit survey, *P* < 0.05

^#^Presents that the part of data missing

Values are presented as mean ± SD

### Hypertension prevalence based on the one-visit survey or D-ABPM

**Fig. 1:**
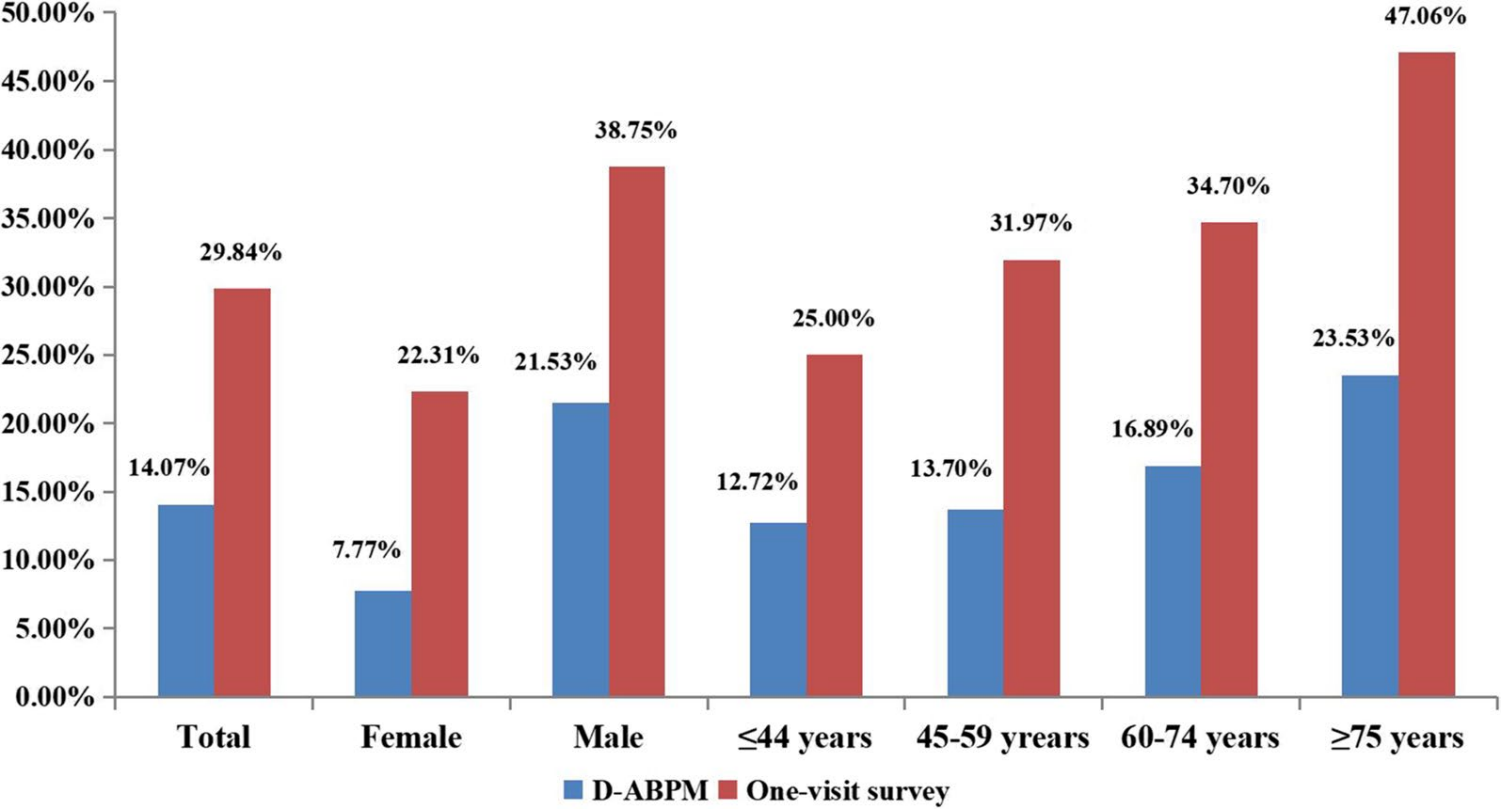
Hypertension prevalence of different screening and measurement patterns

**Fig. 2:**
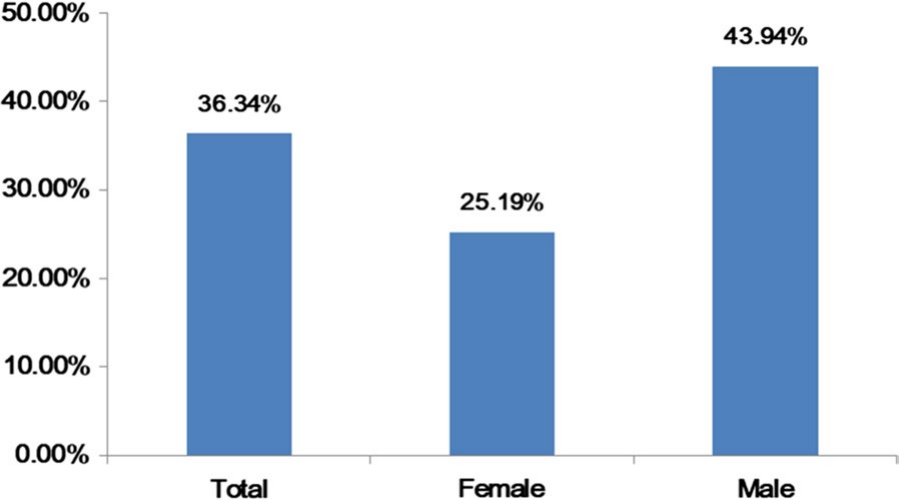
Positive predictive value of one-visit hypertension to predict daytime hypertension

The prevalence of hypertension in the one-visit survey group was 1.12 times higher than that in the D-ABPM group (Fig. 1). Compared with the D-ABPM group, hypertension prevalence in subjects from the one-visit survey group was significantly higher in both males and females, with females displaying a more pronounced trend (Additional file 2: Table S1, Fig. 2).

### PPV of the one-visit survey

We further used the PPV of the one-visit survey to explore the predictive value of one-visit survey in terms of hypertension diagnosis. The PPV of the one-visit survey to hypertension was 36.34% in the total population, 43.94% in males, and 25.19% in females (Fig. 2).

### The demographic and clinical characteristics of the overestimated population

To explore the reasons for such a high prevalence and low PPV of hypertension in the one-visit survey group, this group was further divided into a true positive hypertension group and overestimated group. The true positive group and overestimated group significantly differed in sex, BMI, smoking and drinking status, SBP, DBP, and hypertension subtypes (*P* < 0.05). Most of the overestimated subjects were females, had a BMI < 24.00 kg/m^2^, SBP/DBP < 160/100mmHg, isolated systolic hypertension or isolated diastolic hypertension, and no history of smoking or drinking (*P* < 0.05) (Additional file 2: Table S2).

## Risk factors of the overestimated population

The overestimated hypertension comprised dependent variables. Univariate analyses were conducted using risk factors and traditional hypertension risk factors as independent variables. Multivariate logistic regression analyses were performed after adjusting for demographic and clinical parameters. Risk factors of the overestimated population were female, BMI < 24.00 kg/m^2^, and DBP < 100 mmHg (odds ratio [OR] = 2.42, 95% confidence interval [CI]: 1.33–4.41, *P* < 0.01; OR = 2.55, 95% CI: 1.37–4.77, *P* < 0.01; OR = 20.11, 95% CI: 4.32–93.64, *P* < 0.01) (Table 2).

**Table 2:** The risk factors of overestimated hypertension in one-visit hypertension

Factors	OR	95%CI	*P*
*Sex*			
Male	Reference		
Female	2.42	1.33–4.41	0.004
*BMI groups, (kg/m*^2^)			
≥ 24.00	Reference		
< 24.00	2.55	1.37–4.77	0.003
*DBP groups,(mmHg)*			
≥ 110	Reference		
100–109	2.81	0.54–14.66	0.220
< 100	20.11	4.32–93.64	0.000

CI indicates confidence interval; OR indicates Odds ratio

## The predictive value of SBP/DBP in the one-visit survey for true positive hypertension

**Fig. 3:**
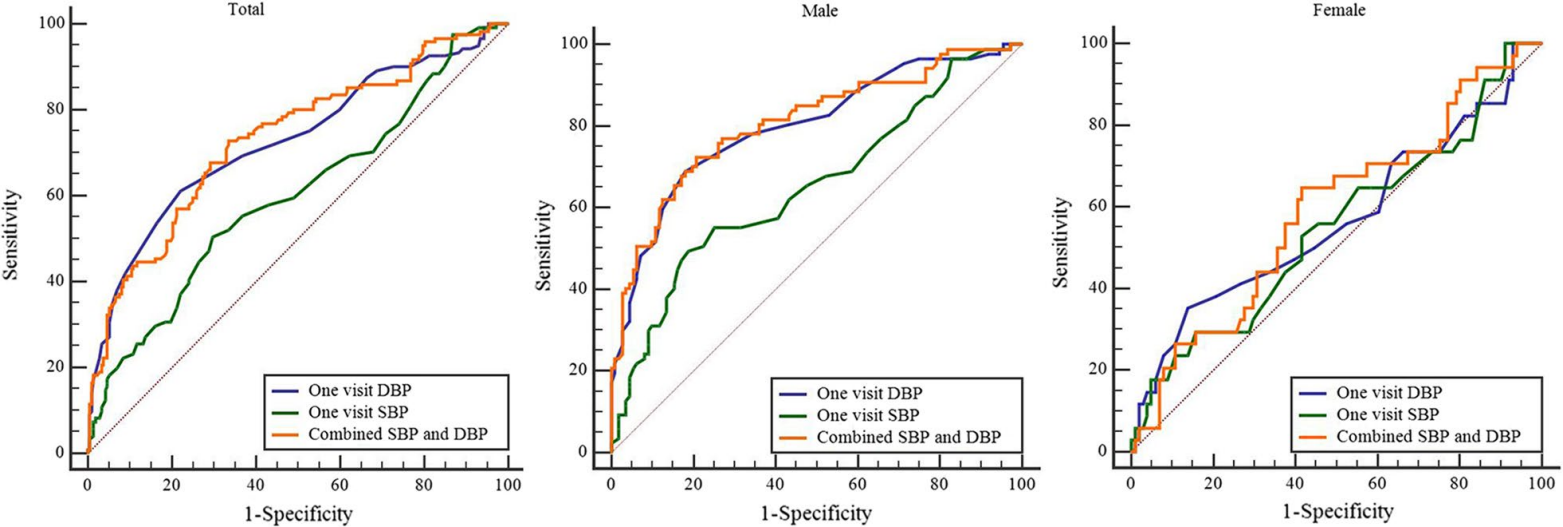
Predictive value of SBP/DBP in one-visit hypertension for true positive hypertension

ROC analysis showed that the AUC of SBP, DBP, and combined SBP and DBP were 0.60, 0.73, and 0.73, respectively. There was no difference between the AUC of the DBP and combination (*P* = 0.661), which were both significantly higher than the AUC of the SBP (*P* < 0.01). The best diagnostic threshold of SBP and DBP was 147.50 and 95.50 mmHg, respectively. In males, the AUC of SBP was 0.65, and the best diagnostic threshold was 147.50 mmHg (49.43% sensitivity, 81.08% specificity). The AUC of DBP was 0.80 and the best diagnostic threshold was 95.50 mmHg (68.97% sensitivity, 81.98% specificity). However, among females, both SBP and DBP in the one-visit survey had no significant predictive value for true positive hypertension (*P* > 0.05) (Table 3 and Additional file 2: Table S3, Fig. 3).

**Table 3:** Predictive value of SBP/DBP in one-visit hypertension for true positive hypertension

Parameters	AUC	95% CI	*P*	Sensitivity (%)	Specificity (%)	Cut-off value
*Total*						
SBP	0.60	0.54–0.67	0.002	50.41	70.28	147.50mmHg
DBP	0.73	0.67–0.79	0.000	61.16	77.83	95.50mmHg
Combination	0.73	0.68–0.79	0.000	72.73	66.51	0.33
*Female*						
SBP	0.55	0.43–0.67	0.397	29.41	84.16	159.50mmHg
DBP	0.57	0.45–0.69	0.247	35.29	86.14	97.50mmHg
Combination	0.59	0.48–0.70	0.128	64.71	58.42	0.32
*Male*						
SBP	0.65	0.58–0.73	0.000	49.43	81.08	147.50mmHg
DBP	0.80	0.74–0.86	0.000	68.97	81.98	95.50mmHg
Combination	0.81	0.75–0.87	0.000	72.41	79.28	0.36

SBP indicates systolic blood pressure; DBP indicates diastolic blood pressure

## Discussion

This study directly compared D-ABPM and the one-visit survey in the same cohort of subjects to evaluate the true prevalence of hypertension. The results showed that hypertension prevalence was likely overestimated by 2-fold in the one-visit survey group. The majority of overestimated subjects were females with DBP < 100mmHg and normal BMI. The characteristics of the overestimated population provided key surveillance objects in the epidemiology of hypertension. In addition, the PPV of hypertension in the one-visit survey group was low. At the same time, this study provides a method to improve the accurate assessment of hypertension in epidemiology, which is of great value to the epidemiology of hypertension prevalence. In males with hypertension diagnosed with the one-visit survey, the diagnostic threshold should be raised to 148/96 mmHg to better diagnose true hypertension. In females, however, the BP measured with the one-visit survey had no significant predictive value for true hypertension. Therefore, it is necessary to further use D-ABPM to determine the prevalence of true hypertension in females. This study assessed the overestimation of hypertension prevalence in a single-visit epidemiological survey, and provided a theoretical basis for the accurate assessment of the prevalence of hypertension in China.

In this study, the prevalence of hypertension in the one-visit survey and D-ABPM groups was 29.84% and 14.07%, respectively. The hypertension prevalence as likely overestimated by 2-fold in the one-visit survey group compared to the D-ABPM group. To the best of our knowledge, very few studies have directly compared the performance of D-ABPM and the one-visit survey. Several studies have found that the prevalence of hypertension may be markedly overestimated when BP is measured during one visit versus two or more visits. In one study, the one-visit survey showed a 1.64-fold increase in the incidence of hypertension compared to the two-visit survey [29]. Figueiredo et al. [11] suggested that the prevalence of hypertension diagnosed during the one-visit survey is overestimated by 12.6% compared to the two-visit survey. A systematic review and meta-analysis of the relative effectiveness of clinic BP measurement and D-ABPM concluded that treatment decisions based on one-visit clinical BP measurement might lead to the overdiagnosis of hypertension [12]. Therefore, hypertension screening should not be performed using the one-visit survey, whereas D-ABPM, which simulates the daytime multiple visit survey, is more advantageous for the screening of hypertension. Asagami [30] showed that D-ABPM performed better than casual BP measurement in terms of repeatability and can provide a repeatable estimate for patients with borderline hypertension. Warren [31] showed that D-ABPM is a more accurate method than the one-visit survey for BP measurement. Rowan [32] found that daytime intensive monitoring lasting at least 6–8 h is required to achieve an effect similar to D-ABPM. The above studies suggest that D-ABPM is superior to the traditional one-visit survey, which is characterized by low accuracy. Our findings suggest that the prevalence of hypertension has been overestimated in multiple surveys, including the May Measurement Month.

Our study demonstrated that the most overestimated cohort was females with DBP < 100 mmHg and normal BMI. We showed that the risk of being overestimated is nearly 20-fold higher for subjects with DBP < 100 mmHg than DBP ≥ 110 mmHg, which indicated that a single BP measurement close to the diagnostic criteria might be inaccurate and overestimated. Therefore, subjects with DBP < 100 mmHg measured by the one-visit survey should be additionally monitored with D-ABPM.

Subjects with a BMI < 24 kg/m^2^ were 2.55-fold more likely to be overestimated than overweight or obese individuals. Others demonstrated that the prevalence of hypertension is higher in overweight or obese subjects than in those with normal weight [33–35]. Overweight or obesity may cause BP to be elevated in a different manner [36]. In obesity, excessive secretion of cytokine leptin by adipocytes promotes renal sympathetic nerve activity (RSNA) by stimulating the central nervous system. Furthermore, the accumulation of fat in the viscera, perirenal space, and renal sinuses could lead to kidney compression and thus activate the renin-angiotensin-aldosterone system in addition to RSNA. Adipocytes may also activate mineralocorticoid receptors independent of aldosterone to further elevate BP. In addition, overweight/obese subjects are often characterized by renal insufficiency, abnormal lipid metabolism and insulin resistance, which together exacerbates the hypertension [37]. Thus, overweight or obese individuals tend to be true positive for hypertension, whereas those with normal BMI are at high risk of being overestimated.

This study found that females were 2.42-fold more likely to be overestimated than males. It is generally believed that males have a higher risk of hypertension than females, which could be explained by their mental stress as well as unhealthy habits such as smoking, drinking, overworking, intake of high-fat products, and lack of exercise [38–41]. Our results suggest that males tend to be true positive for hypertension, whereas females tend to be overestimated. In conclusion, females with DBP < 100 mmHg and BMI < 24.00 kg/m^2^ are more likely to be misdiagnosed, which further requires D-ABPM to determine true positive hypertension.

This study showed that the PPV of hypertension in one population visit survey was only 36.34%. A previous study evaluated the PPV of the one-visit BP measurement by comparing the prevalence of hypertension in one- and two-visit surveys, and showed that the hypertension PPV for the one-visit survey was 41.0% [28], consistent with our results. Such low PPV of one-visit surveys may reduce the accuracy of hypertension screening. Therefore, it is important to re-establish the diagnostic threshold for those diagnosed with positive hypertension during the one-visit survey. Our findings suggest that the threshold of hypertension should be raised to 148/96 mmHg for the more accurate diagnosis of hypertension in males. However, in females, BP in the one-visit survey had no significant predictive value for true positive hypertension.

This study had several advantages. Few studies have used D-ABPM to screen for hypertension prevalence in the general population. In this study, we evaluated the overestimation of the hypertension prevalence by the one-visit survey. Furthermore, we suggested how to effectively identify the overestimated population by providing a new BP diagnostic threshold. However, this study has several limitations. First, although ABPM is considered the gold standard for the diagnosis of hypertension, the common practice is performing 3 blood pressure measurements in different days. Moreover, some studies still suggest that ABPM may not completely replace the repeated blood pressure measurements in the clinic [42]. Second, subjects without history of hypertension were enrolled in this study in a continuous way instead of a random way, which may have an influence on the prevalence of hypertension. Furthermore, all the participants of this study were from Hebei Province, which may not represent the entire country. We will address these limitations in our future large-scale, multi-center studies to reflect the prevalence of hypertension more accurately.

## Conclusion

The mean value of BP in the one-visit survey group was about 8 mmHg higher than that in the D-ABPM group, and the hypertension prevalence was likely to be twice as overestimated in the one-visit survey group. Most of the overestimated subjects were females with DBP < 100 mmhg and normal BMI. The PPV of hypertension in the one-visit survey group was low. In males with hypertension diagnosed by the one-visit survey, the diagnostic threshold should be raised to 148/96 mmHg to better diagnose true positive hypertension. However, in females, BP in the one-visit survey had no significant predictive value for true positive hypertension. Therefore, it is necessary to further perform D-ABPM in females to determine the true hypertension.

## Supplementary Information

**Additional file 1.** **Supplementary Figure S1.** A schematic illustrating the inclusion/exclusion of the participants.

**Additional file 2.** Supplementary Tables incuding Table S1, Table S2 and Table S3.

## Data Availability

The datasets generated during and/or analyzed during the current study are available from the corresponding author on reasonable request.

## Abbreviations

ABPM: 24-hour ambulatory blood pressure monitoring
D-ABPM: Daytime ambulatory blood pressure monitoring
BP: Blood pressure
SBP: Systolic blood pressure
DBP: Diastolic blood pressure
BMI: Body mass index
CHOL: Cholesterol
eGFR: Estimation of glomerular filtration rate
CI: Confidence interval
OR: Odds ratio
