# Supplementary figures and images for: The overestimated prevalence of hypertension in a population survey: a cross-sectional study from Hebei province, China
Source: BMC Cardiovasc Disord. 2022 Dec 12;22:542. doi: 10.1186/s12872-022-02994-y (PMC9743587; doi:10.1186/s12872-022-02994-y)

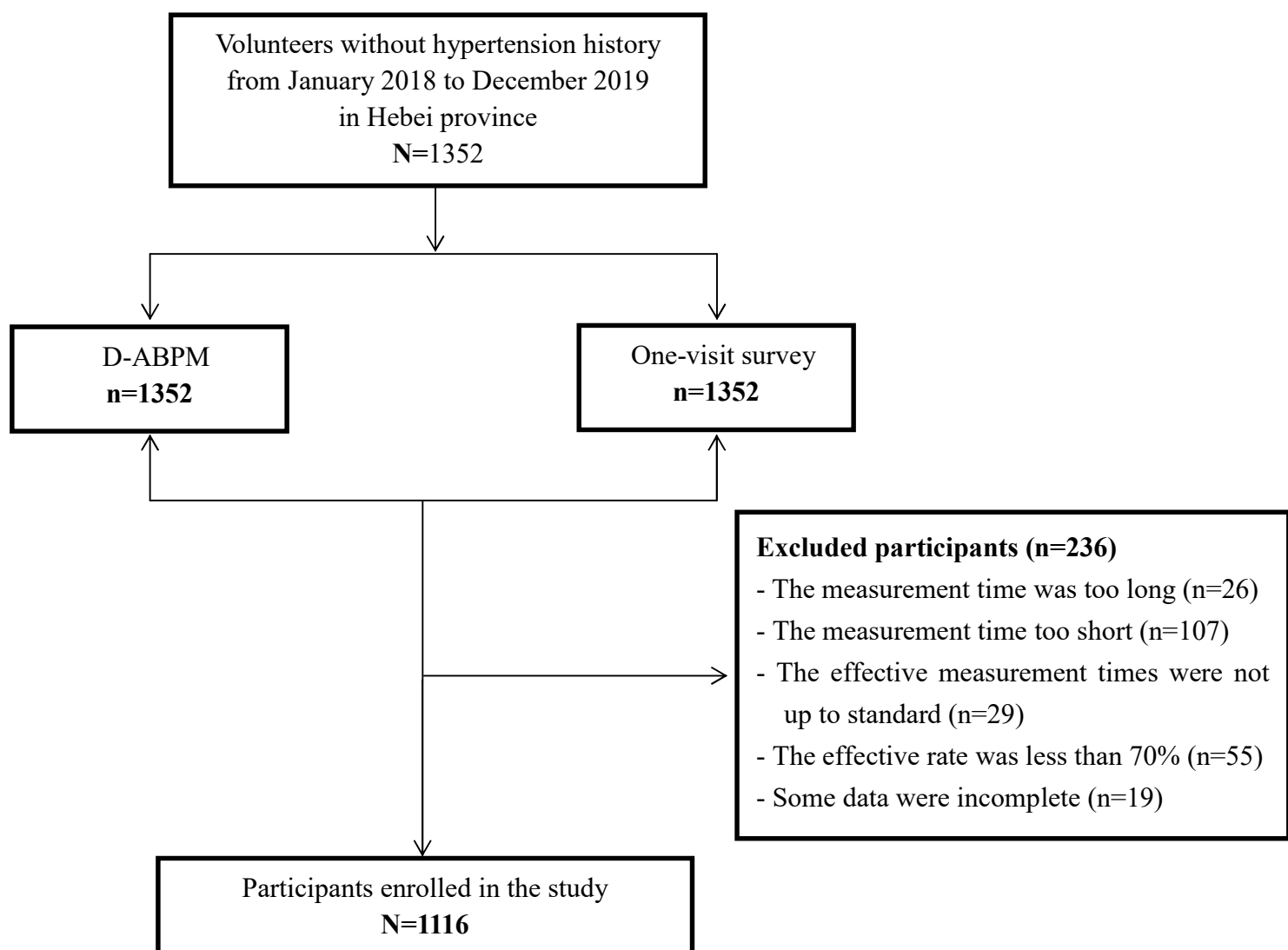

Supplement: Supplementary file 1 — Additional file 1. Supplementary Figure S1. A schematic illustrating the inclusion/exclusion of the participants. [file 12872_2022_2994_MOESM1_ESM.pdf]
