# Supplementary material for: The overestimated prevalence of hypertension in a population survey: a cross-sectional study from Hebei province, China
Source: BMC Cardiovasc Disord. 2022 Dec 12;22:542. doi: 10.1186/s12872-022-02994-y (PMC9743587; doi:10.1186/s12872-022-02994-y)
Supplement: Supplementary file 2 — Additional file 2. Supplementary Tables incuding Table S1, Table S2 and Table S3. [file 12872_2022_2994_MOESM2_ESM.doc]

Supplementary Figure Legend

Figure S1. A schematic illustrating the inclusion/exclusion of the participants

Table S1. Hypertension prevalence of different screening and measurement patterns

| Population | Multiple visit hypertension  (D-ABP≥135/85mmHg) | One-visit hypertension  (BP≥140/90mmHg) | *P* |
| --- | --- | --- | --- |
| Total(1116) | 14.07%(157/1116) | 29.84% (333/1116) | 0.000 |
| Sex |  |  |  |
| Female(605) | 7.77% (47/605) | 22.31% (135/605) | 0.000 |
| Male(511) | 21.53% (110/511) | 38.75% (198/511) | 0.000 |
| Age groups, (years) |  |  |  |
| ≤44(464) | 12.72% (59/464) | 25.00% (116/464) | 0.000 |
| 45~59(416) | 13.70% (57/416) | 31.97% (133/416) | 0.000 |
| 60~74(219) | 16.89% (37/219) | 34.70% (76/219) | 0.000 |
| ≥75(17) | 23.53% (4/17) | 47.06% (8/17) | 0.294 |
| Note: BP indicates blood pressure; D-ABP indicates daytime ambulatory blood pressure | | | |

Table S2. The demographic and clinical characteristic of one-visit hypertension

| Factors | | | Overestimated Hypertension  (n=212) | True positive hypertension  (n=121) | *P* |
| --- | --- | --- | --- | --- | --- |
| Sex | | |  |  | 0.000 |
|  | Female | | 101(74.80%) | 34(25.20%) |  |
|  | Male | | 111(56.10%) | 87(43.90%) |  |
| Age groups, (years) | | |  |  | 0.992 |
|  | ≤44 | | 73(62.90%) | 43(37.10%) |  |
|  | 45-59 | | 86(64.70%) | 47(35.30%) |  |
|  | 60-74 | | 48(63.20%) | 28(36.80%) |  |
|  | ≥75 | | 5(62.50%) | 3(37.50%) |  |
| BMI group, (kg/m^2^) | | |  |  | 0.000 |
|  | <24.00 | | 83(76.90%) | 25(23.10%) |  |
|  | ≥24.00 | | 127(57.00%) | 96(43.00%) |  |
| Occupation | | |  |  | 0.076 |
|  | Physical laborer | | 83(63.80%) | 47(36.20%) |  |
|  | Staff | | 83(62.40%) | 44(34.60%) |  |
|  | Retirees | | 28(57.10%) | 21(42.90%) |  |
|  | Freelance | | 18(66.70%) | 9(33.30%) |  |
| Smoking | | |  |  | 0.009 |
|  | No | | 141(69.10%) | 63(30.90%) |  |
|  | Yes | | 71(55.00%) | 58(45.00%) |  |
| Drinking | | |  |  | 0.010 |
|  | No | | 134(69.40%) | 59(30.60%) |  |
|  | Yes | | 78(55.70%) | 62(44.30%) |  |
| Medical history: n (%) | | |  |  |  |
| Hyperlipidemia history | | |  |  | 0.773 |
|  | No | | 198(63.90%) | 112(36.10%) |  |
|  | Yes | | 14(60.90%) | 9(39.10%) |  |
| Diabetes history | | |  |  | 0.763 |
|  | No | | 203(63.80%) | 115(36.20%) |  |
|  | Yes | | 9(60.00%) | 6(40.00%) |  |
| Cardiovascular history | | |  |  | 0.496 |
|  | No | | 205(63.30%) | 119(36.70%) |  |
|  | Yes | | 7(77.80%) | 2(22.20%) |  |
| Cerebrovascular history | | |  |  | 1.000 |
|  | No | | 207(63.50%) | 119(36.50%) |  |
|  | Yes | | 5(71.40%) | 2(28.60%) |  |
| Respiratory history | | |  |  | 0.104 |
|  | No | | 210(64.40%) | 116(35.60%) |  |
|  | Yes | | 2(28.60%) | 5(71.40%) |  |
| Chronic renal damage | | |  |  | 0.131 |
|  | No | | 212(64.00%) | 119(36.00%) |  |
|  | Yes | | 0(0.00%) | 2(100.00%) |  |
| CHOL groups, (mmol/L) | | |  |  | 0.494 |
|  | <3.11 | | 69(68.30%) | 32(31.70%) |  |
|  | ≥3.11~<5.20 | | 91(61.90%) | 56(38.10%) |  |
|  | ≥5.20 | | 40(60.60%) | 26(39.40%) |  |
| eGFR groups,(ml/min/1.73m^2^) | | | |  | 0.271 |
|  | ≥90 | 167(65.00%) | | 90(35.00%) |  |
|  | <90 | 29(56.90%) | | 22(43.10%) |  |
| SBP groups, (mmHg) | |  | |  | 0.002 |
|  | <160 | 187(67.50%) | | 90(32.50%) |  |
|  | 160-179 | 24(47.10%) | | 27(52.90%) |  |
|  | ≥180 | 1(20.00%) | | 4(80.00%) |  |
| DBP groups, (mmHg) | |  | |  | 0.000 |
|  | <100 | 197(72.40%) | | 75(27.60%) |  |
|  | 100-109 | 13(28.90%) | | 32(71.10%) |  |
|  | ≥110 | 2(12.50%) | | 14(87.50%) |  |
| Hypertension subtypes | |  | |  | 0.000 |
|  | Non-HP | 0(0.00%) | | 0(0.00%) |  |
|  | ISH or IDH | 139(73.20%) | | 51(26.80%) |  |
|  | SDH | 73(51.00%) | | 70(49.00%) |  |
| Note: BMI indicates body mass index; CHOL indicates cholesterol; SBP indicates systolic blood pressure; DBP indicates diastolic blood pressure; eGFR indicates estimation of glomerular filtration rate; HP indicates hypertension; ISH indicates isolated systolic hypertension; IDH indicates isolated diastolic hypertension; SDH indicates systolic-diastolic hypertension | | | | | |

Table S3. The comparison of Area under ROC curve

| Parameters | Z | *P* |
| --- | --- | --- |
| Total |  |  |
| Combination-DBP | 0.44 | 0.661 |
| Combination-SBP | 3.98 | 0.000 |
| DBP-SBP | 2.95 | 0.003 |
| Female |  |  |
| Combination-DBP | 0.70 | 0.483 |
| Combination-SBP | 0.52 | 0.601 |
| DBP-SBP | 0.19 | 0.849 |
| Male |  |  |
| Combination-DBP | 0.55 | 0.584 |
| Combination-SBP | 4.21 | 0.000 |
| DBP-SBP | 3.14 | 0.002 |
| Note: SBP indicates systolic blood pressure; DBP indicates diastolic blood pressure | | |
